# Supplementary material for: Intestinal Microbiota in Children with Anemia in Southern Peru through Next-Generation Sequencing Technology
Source: Children (Basel). 2022 Oct 25;9(11):1615. doi: 10.3390/children9111615 (PMC9688611; doi:10.3390/children9111615)
Supplement: Supplementary file 1 [file children-09-01615-s001.zip › children-1851337-supplementary.pdf]

Intestinal Microbiota in Children with Anemia in Southern Peru Through Next-Generation Sequencing Technology

Karla Díaz-Rodríguez [1], Jani Pacheco-Aranibar [1,3], Cecilia Manrique-Sam [1], Yuma Ita-Balta [1], Agueda Muñoz-del-Carpio-Toia [1], Patricia López-Casaperalta [1,4], Teresa Chocano-Rosas [1], Fernando Fernandez-F [1], Jose Villanueva-Salas [1] and Julio Cesar Bernabe-Ortiz [1,3,*]

[1] Universidad Católica de Santa María, Post-Graduate School, Urb. San José s/n, Umacollo, Arequipa 04013, Peru

[2] Universidad Nacional de San Agustín. Arequipa – Perú.
[3] Instituto de Biotecnologia del ADN Uchumayo. Arequipa – Perú;
[4] Universidad Católica de Santa María, Urb. San José s/n, Umacollo, Arequipa 04013, Peru

* Correspondence: jbernabe@ucsm.edu.pe (J.C.B.-O.)

Supplementary Figures

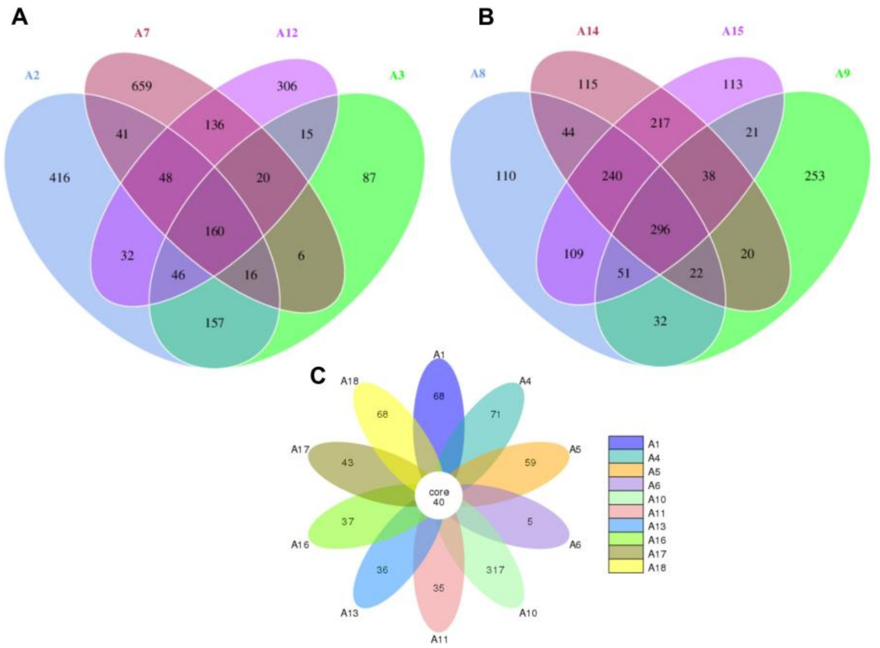

Figure S1. Venn and Flower diagram of (A) Control children, (B) Recovered children, and (C) Children with anemia.

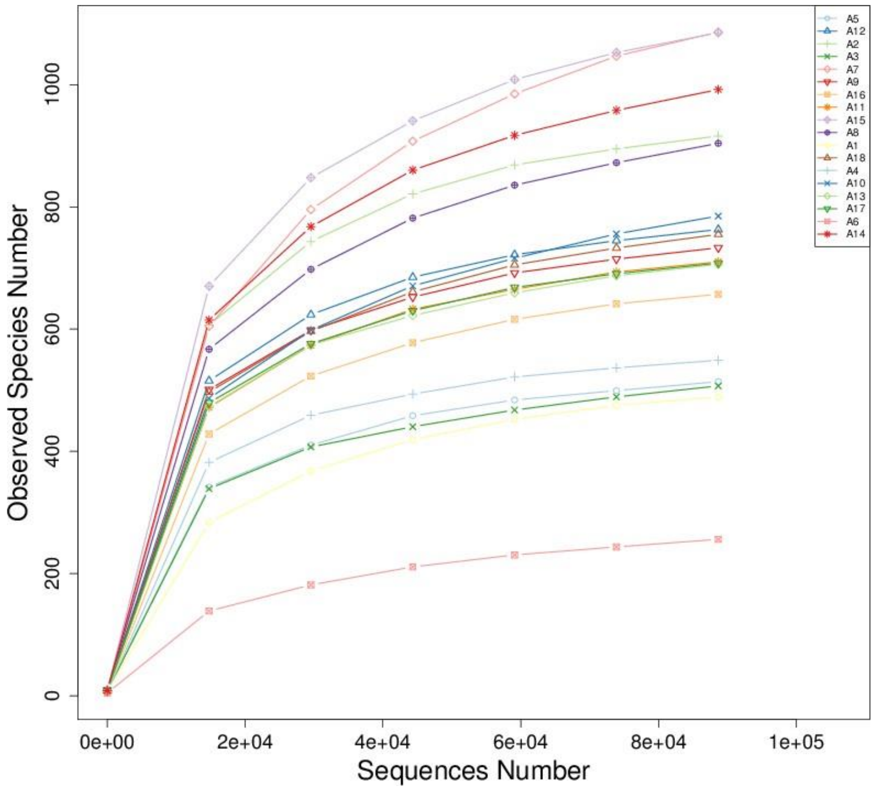

Figure S2. Rarefaction curve. The y-axis shows the number of species observed, while the x-axis indicates the number of sequences. Each sample from A1 to A18 is represented with a different color. The curve suggests that the libraries of the samples included in this study were sufficient to capture most of the bacterial diversity.
